# Stable isotopes provide evidence that condensed tannins from sericea lespedeza are degraded by ruminal microbes

**DOI:** 10.1038/s41598-022-18566-1

**Published:** 2022-08-22

**Authors:** Flavia O. S. van Cleef, José C. B. Dubeux, Chrisee S. Wheeler, Carlos C. V. García, Martin Ruiz-Moreno, Lynn E. Sollenberger, João M. B. Vendramini, Nicolas DiLorenzo, Harley D. Naumann

**Affiliations:** 1 Agronomy Department, North Florida Research and Education Center, University of Florida, Marianna, FL 32446 USA; 2 Division of Plant Sciences, University of Missouri, Columbia, MO 65201 USA; 3 Agronomy Department, University of Florida, Gainesville, FL 32611 USA; 4 Agronomy Department, Range Cattle Research and Education Center, University of Florida, Ona, FL 33865 USA

**Keywords:** Climate-change mitigation, Secondary metabolism

## Abstract

The objective of Trial 1 was to determine the effects of condensed tannins (CT) from sericea lespedeza [SL; *Lespedeza cuneata* (Dum. Cours.) G. Don] on in vitro digestible organic matter (IVDOM), total gas production (GP), methane (CH_4_) emission, and ruminal fluid parameters after fermentation. Substrates used in four 48-h in vitro fermentations were 100% bermudagrass [(*Cynodon dactylon* (L.) Pers.] hay (0SL), 100% SL hay (100SL), and a mix of both hays (50SL). Linear reductions were observed for all parameters (*P* < 0.05) with the inclusion of SL, except for CH_4_ in relation to GP, that presented a quadratic effect (*P* = 0.005). In Trial 2, SL plants were enriched with ^13^C–CO_2_ to obtain pure enriched CT to identify the destination of fermentation end products of CT degradation. The enrichment of CT through the SL was successful (*P* < 0.001), and carbon originated from CT was detected in the fermentation end products [microbial mass, clarified rumen fluid, and in the CH_4_ produced (*P* < 0.001)]. Therefore, inclusion of SL was effective in reducing in vitro CH_4_ production and compound-specific tracing of δ^13^C abundance provided better quantitative understanding of the mechanisms of partitioning CT during ruminal fermentation processes.

## Introduction

Condensed tannins (CT) are specialized plant metabolites that have been widely studied in human nutrition^1^, and are commonly used in the diets of ruminants as natural mediators of rumen fermentation^2–4^. Some tannin-rich legumes, such as sericea lespedeza [SL; *Lespedeza cuneata* (Dum. Cours.) G. Don], are of particular interest because of their anti-helminthic and anti-bloat properties and the potential for mitigation of enteric CH_4_ emissions similar to that of ionophores and vegetable oils^5^.

Although it is generally assumed that CT are not degraded or absorbed^6,7^, many studies have reported less than 100% of recovery of tannins in feces and in digesta^8–10^. van Cleef et al.^11^ fed beef steers a tannin-containing forage and evaluated CT concentration in feces using the butanol-HCl assay^12^. In addition to a partial recovery of CT compared to what was originally fed as forage, the fecal CT determined was entirely bound to fiber or protein fractions. Such inefficiency in recovery results in contradictory findings, due to either an analytical limitation or to inconsistency in research aiming to determine the kinetics and fate of tannins. The structural diversity of CT and their challenging chemical determination are the greatest limitations in advancing knowledge to elucidate their effects on kinetics in ruminant nutrition^13^.

Isotopic labeling of plants has been useful in tracking the fate of carbon from different plant parts in a soil–plant system^14,15^. However, structural components of the enriched plant material can present different microbial utilization and long-term carbon storage than that found in metabolic components^16,17^. Thus, compound-specific tracing of δ^13^C abundance could provide a novel and powerful tool for a better quantitative understanding of the mechanisms of partitioning CT molecules undergoing ruminal fermentation processes.

We hypothesized in Trial 1 that SL as sole source of feed for ruminal microorganisms would present lesser in vitro OM digestibility than the grass-based substrate, and CH_4_ production could be reduced due to the presence of CT. In Trial 2, the hypothesis was that labeling SL with ^13^C-enriched CO_2_ would enrich ^13^C in the molecules of CT as well. Thus, it would be possible to trace the carbon in CT from SL used during fermentation by ruminal microorganisms because of the fractionation of ^13^C among different products of fermentation. Therefore, the major objectives of this study were to determine the in vitro digestibility and CH_4_ production associated with consumption of SL hay and assess possible CT digestion by ruminal microorganisms.

## Results

### Trial 1

The effects of inclusion of sericea lespedeza (SL) on fermentation parameters are described in Table 1. The inclusion of SL linearly reduced most of the parameters evaluated, except for pH and CH_4_ in relation to gas production (GP), which were quadratically affected (*P* < 0.001 and *P* = 0.005, respectively). Isotopic composition of CH_4_ was also linearly affected (*P* = 0.006), with CH_4_ from substrate 100SL being the most depleted in ^13^C (δ^13^C = − 51.33‰).

**Table 1:** Forty-eight-hour in vitro fermentation parameters of three proportions of sericea lespedeza [SL; *Lespedeza cuneata* (Dum. Cours.) G. Don] hay with bermudagrass[*Cynodon dactylon* (L.) Pers.] hay diets from Trial 1.

Item	Treatments^a^			SEM	*P* value	
	0SL	50SL	100SL		L	Q
pH	6.63	6.67	6.77	0.011	< 0.001	< 0.001
NH_3_–N, mM	1.18	1.15	1.10	0.031	0.001	0.076
GP, mL/g OM	59.45	49.89	36.14	7.678	< 0.001	0.071
IVDOM, %	41.14	38.49	33.31	1.956	< 0.001	0.061
GP/IVDOM, mL/g	1.44	1.29	1.08	0.216	< 0.001	0.468
Total CH_4_, mL/g OM	2.83	1.68	1.34	0.708	0.006	0.109
CH_4_/GP, %	4.76	3.37	3.71	0.041	0.004	0.005
CH_4_/IVDOM, mL/g	6.88	4.36	4.02	1.75	0.025	0.305
δ^13^C–CH_4_, ‰	− 42.61	− 42.94	− 51.33	3.012	0.006	0.917

GP, gas production; IVDOM, in vitro digestible organic matter; NH_3_–N, ammonia–nitrogen; CH_4_, methane; SEM, standard error of the mean; L, linear effect of SL inclusion; Q, quadratic effect of SL inclusion.

^a^0SL: 0% (fresh weight basis) sericea lespedeza hay (100% bermudagrass hay); 50SL: 50% sericea lespedeza hay + 50% bermudagrass hay; 100SL: 100% sericea lespedeza hay (0% bermudagrass hay).

Volatile fatty acids (VFA) molar proportions are represented in Table 2. Total VFA concentration and acetate:propionate ratio decreased linearly as inclusion of SL increased (*P* = 0.031 and *P* = 0.003, respectively). Molar proportions of acetate, propionate, and butyrate were quadratically affected (*P* = 0.001, *P* = 0.032, and *P* < 0.001, respectively) by the inclusion of SL, whereas molar proportions of BCVFA were not affected.

**Table 2:** Total volatile fatty acids (VFA) and VFA profile of in vitro fermentation from Trial 1.

Item	Treatments^a^			SEM	*P* value	
	0SL	50SL	100SL		L	Q
Total VFA, m*M*	69.7	63.3	50.2	5.76	0.031	0.081
A:P^b^	3.36	3.03	3.14	0.213	0.003	0.560
**VFA molar proportions, mol/100 mol**						
Acetate	70.3	69.8	72.1	1.08	0.173	0.001
Propionate	20.9	23.0	22.9	1.02	0.001	0.032
Butyrate	6.7	5.7	3.5	0.13	< 0.001	< 0.001
BCVFA	1.2	1.4	1.0	0.17	0.461	0.075

SEM, standard error of the mean; L, linear effect of SL inclusion; Q, quadratic effect of SL inclusion; BCVFA, branched-chain volatile fatty acids = isobutyrate + isovalerate + 2-methylbutyrate.

^a^SL: sericea lespedeza [*Lespedeza cuneata* (Dum. Cours.) G. Don]  0SL: 0% (fresh weight basis) SL hay (100% bermudagrass hay); 50SL: 50% SL hay + 50% bermudagrass hay; 100SL: 100% SL hay (0% bermudagrass hay).

^b^Acetate:propionate ratio.

### Trial 2

There were effects of substrates on terminal pH (*P* = 0.002; SEM = 0.032), with pure CT extracts presenting the greatest pH values (6.83 and 6.84 for Enriched CT and Not Enriched CT, respectively). Substrates of whole plants (SL Enriched, Alfalfa, and SL Not Enriched) presented lesser pH than CT-only substrates (6.76, 6.68, 6.72, respectively) but did not differ from substrates containing alfalfa mixed with CT, regardless of enrichment (6.76 for both).

**Figure 1:**
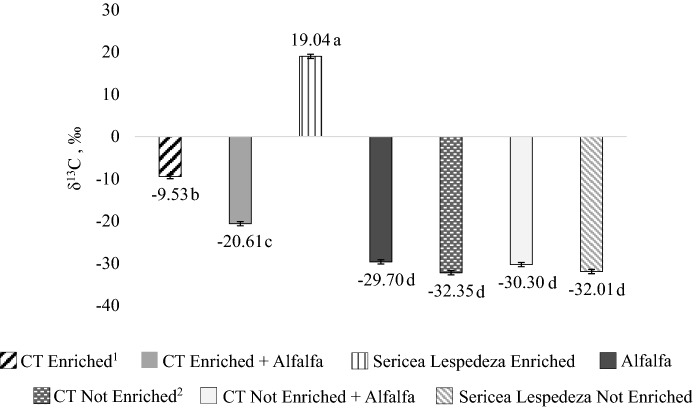
Isotopic composition of substrates used on in vitro incubations of Trial 2 determined with isotope ratio mass spectrometry. ^1^CT enriched: condensed tannins enriched with ^13^C–CO_2_. ^2^CT not enriched: condensed tannins not enriched with ^13^C–CO_2_. Means with different letters are significantly different by Tukey test (*P* < 0.05). Bars refer to the standard error.

The uniform labeling of SL and of the CT molecule was effective and is detailed in Fig. 1 by the differences in the δ^13^C composition of the substrates (*P* < 0.001). The enriched plant of SL was more enriched in its δ^13^C composition than the non-enriched SL. Likewise, the CT extract from the enriched SL plants was more enriched in ^13^C than CT extract from non-enriched plants (*P* = 0.002). Alfalfa used in this trial presented a δ^13^C of − 29.70‰.

**Figure 2:**
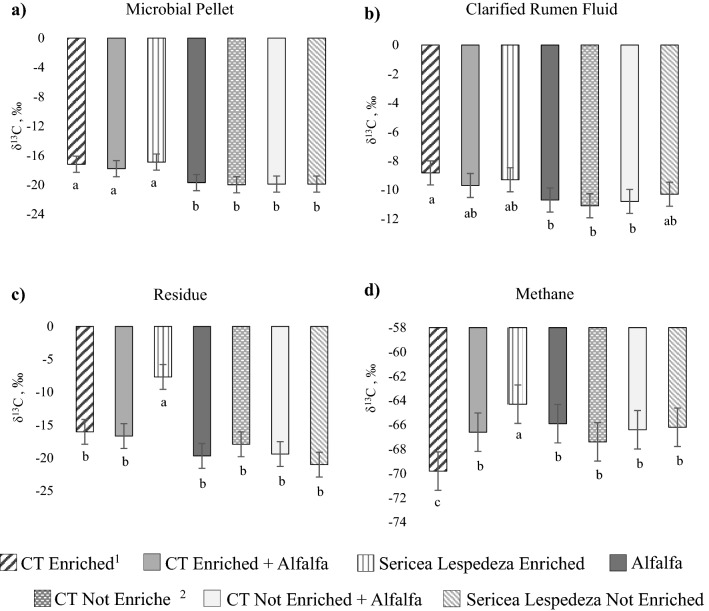
Effects of enrichment of sericea lespedeza [*Lespedeza cuneata *(Dum. Cours.) G. Don] with ^13^C–CO_2_ on (**a**) microbial pellet, (**b**) clarified rumen fluid, (**c**) residue pellet, and (**d**) methane from in vitro fermentation from Trial 2. Isotopic composition of end-products of fermentation determined with isotope ratio mass spectrometry. ^1^CT enriched: condensed tannins enriched with ^13^C–CO_2_. ^2^CT not enriched: condensed tannins not enriched with ^13^C–CO_2_. Means with different letters are significantly different by Tukey test (*P* < 0.05). Bars refer to the standard error.

The δ^13^C of end products of fermentation are listed in Fig. 2. The δ^13^C composition of the microbial pellet of CT Enriched, CT Enriched + Alfalfa, and SL Enriched differed from Alfalfa, CT Not Enriched, CT Not Enriched + Alfalfa and SL Not Enriched (Fig. 2a; *P* < 0.001). Clarified rumen fluid δ^13^C composition from CT Enriched was the least depleted in ^13^C (Fig. 2b), differing from Alfalfa, CT Not Enriched, and CT Not Enriched + Alfalfa (*P* = 0.001). The δ^13^C of residue of fermentation (Fig. 2c) of SL Enriched was the least depleted substrate and differed from all others (*P* < 0.001). Methane produced by SL Enriched was the least depleted in ^13^C (Fig. 2d). Conversely, CH_4_ produced by CT Enriched was more depleted in ^13^C than all non-enriched substrates (*P* < 0.0001).

When determining the proportions of C originated from CT (Table 3), the enriched and not enriched substrates differed in microbes (*P* < 0.001), clarified rumen fluid (*P* < 0.001) and CH_4_ gas (*P* < 0.001).

**Table 3:** Contribution of carbon from condensed tannins (CT) in enriched or not-enriched mixed substrates from in vitro fermentation from Trial 2.

% C from CT	Product of fermentation			
	Microbes	Clarified rumen fluid	Residue	Methane
CT enriched + Alfalfa	55.1	88.5	66.9	88.3
CT not enriched + Alfalfa	68.4	68.6	68.2	66.5
Statistics*				
*P* value	< 0.001	< 0.001	0.710	< 0.001
SEM	1.51	1.63	3.98	0.43

*Means are considered different when *P* < 0.05.

## Discussion

Previous studies in which ruminants were fed forage legumes with CT mainly focused on the interactions between the phenol and dietary constituents, particularly proteins. However, the fate of CT as they move along the digestive tract of ruminants is still unclear. This is mainly due to low CT concentrations found in digesta and low recovery rates in feces, while there has been no direct evidence of the degradation or absorption of CT. Additionally, growth conditions of the plants affect not only the synthesis of CT but its concentration and bioactivity, as well as the chemical composition of the whole plant, which influences the fermentability and potential to mitigate CH_4_. Thus, Trial 1 brings in vitro results of digestibility and CH_4_ production to complement existing information in the literature about SL. In the more in-depth investigation, Trial 2 brings a novel approach to determine the origin of the carbon in the fermentation end products and ultimately that the carbon from CT was altered by ruminal microorganisms. Despite methodological limitations imposed by chemical determination of CT, this partially justifies the lack of detection of the molecule in feces and digesta^10,11^.

In Trial 1, the inclusion of SL was effective in reducing the total gas produced, which is an indicator of slowed fermentation and rumen microbial growth^18^. In general, diets that provide a lower digestion rate produce less gas and consequently increase pH of the ruminal fluid^19^, as observed in the current study for substrate 100SL. However, the inhibitory effect of CT-protein complexes on the rumen fibrolytic bacteria may have affected the degradation of the cell wall and reduced the IVDOM when 100% SL was included^20,21^, even at greater levels of CP. Thus, it is unlikely that any nutritional benefits resulted from greater levels of CP intake when digestibility of nutrients and other plant components were compromised^22^.

Our findings of decreased gas production with the inclusion of 100% SL likely suggests that fermentation was suppressed, which affected the extent of OM degradation. Consequently, there was a reduction of total CH_4_ and CH_4_ per unit of digested OM, possibly because less nutrients were available for ruminal microorganisms, but also because CT present in SL might have directly inhibited their activity^13^. Moreover, CTs from SL are richer in prodelphinidin (PD) than procyanidin (PC) flavan-3-ol units^22^, which are reported to consist of larger polymers and likely bind more tightly to proteins and fiber. Thus, in a digestion process, we hypothesize that PD-rich CT would degrade at a slower rate and possibly to a lesser extent compared to PC-rich CT. Consequently, SL may be less digested than other legumes containing other types of CT (i.e., *Lotus corniculatus*).

Lesser total VFA concentration is also a reflection of lesser cell wall degradation and reduced IVDOM^23,24^. This decrease in total VFA concentrations in rumen fluid from substrates 50SL and 100SL agrees with the negative effect of moderate to high CT concentrations on rumen carbohydrate degradation and VFA production commonly observed in vivo and in vitro^7,25,26^.

Studies have shown that CT shift carbohydrate fermentation towards higher propionate and lower acetate production^25^, accompanied by a reduction in ammonia absorption from the rumen^27^. Ruminal VFA concentrations and in vitro CH_4_ production are strongly correlated with the acetate:propionate ratio, which is dependent upon the pH and substrate nature^28^, mainly because an increase in propionate have been associated with increased protein flow from the rumen^29^. Nonetheless, the reduction observed in our study in total VFA concentration is mainly due to the decrease in butyrate proportion and not to an increase in propionate.

Fermentation of amino acids (valine, isoleucine, leucine, and proline) and microbial deamination can affect the ruminal pH^30–32^. However, because concentration of ammonia in the rumen is a function of ammonia production, ammonia uptake by the ruminal microorganisms, and diffusion through the rumen wall, in in vitro studies there is limited uptake of ammonia by ruminal microorganisms under lower pH, and no diffusion through the rumen wall^33^, which favors accumulation of ammonia in the bottle. However, with the presence of CT from substrates containing SL, there was a reduction in ammonia concentrations, demonstrating the binding ability of CT to proteins. Thus, because less protein was being degraded in ruminal conditions, less total VFA was produced by substrates 50SL and 100SL^34–36^.

Isotopic fractionation is a result of digestion, absorption, assimilation, and excretion of nutrients^37^, and because C_3_ and C_4_ plants differ in the pathways of carbon fixation, it was possible to determine differences in nutritive value of SL and bermudagrass by their isotopic composition^38,39^. In the current study, there was lesser increment in the discrimination of ^13^C from 0 to 50SL, but greater discrimination of ^13^C occurred when SL was added at 100%. The more depleted CH_4_ from 100SL revealed the least in vitro OM digestibility, whereas the most enriched CH_4_ was found for 0SL, whose OM digestibility was the greatest.

A great challenge of Trial 2 was to determine if any carbon from CT would be metabolized by ruminal microorganisms during fermentation, regardless of the extent of fermentation of each substrate. For this to occur, the labelling of SL for short times (1 h, for 10 days, every 30 days) would have to be sufficient for the ^13^C to reach the molecule of CT through natural discrimination. Natural discrimination is a result of kinetic fractionation and isotope distribution when plants synthesize compounds that become more depleted than the primary compounds, such as carbohydrates^16^. Thus, the CT extracted from SL is more depleted in ^13^C than the original SL plant material due to this natural discrimination. We determined that, consequently, a tannin-rich plant that has been enriched with ^13^C-CO_2_ presented CT and other natural compounds more enriched in ^13^C than a non-enriched plant.

Although there was no direct evidence of the degradation and absorption of CT in the digestive tract of ruminants until now, many studies reported quantitatively less intact CT were excreted than were ingested^9–11,40,41^. However, possible methodological issues of common assays could hinder the detection of CT in feces. The color reaction expected in the butanol-HCl assay^12^ is dependent on the interflavan bond type and the recalcitrance of the interflavan bond. In Naumann et al.^42^, for example, *Acacia angustissima* used had very stable interflavan bonds, and the color development was weak, resulting in an underestimation of the CT.

The presence of tannin-protein complexes in fiber fractions was found to be similar to lignin in feces^43^ and difficulties were reported in determining CT in feces using the aforementioned assay^44^. However, Perez-Maldonado and Norton^41^ suggested an absorption of the CT or some bacterial degradation throughout the gastrointestinal tract to explain the lack of CT in feces.

Herewith, by labelling our plants of SL with ^13^C-CO_2_ we determined that carbon from its CT was present in the end products of fermentation, including the biomass of microbes, that is actively engaged in fermentation of carbohydrates and proteins. Furthermore, the prediction of CT contribution in most fermentation end products, except for the solid residue, reveals that more than 50% of the carbon found in those end products came from CT, while the rest of carbon originated from the other legume, alfalfa. Thus, the carbon from the molecule of CT from SL was used and discriminated by ruminal microbes during fermentation of substrates even in the absence of other substrates.

When isotopically fractionating processes happen, the substrate and the product have a variation in the isotopic ratios. The greater depletion of the CH_4_ produced by the enriched substrates is related to the action of the enzymes involved in the process of ^13^C fractionation, due to their specific role in the pathway of fermentation^45^. Although an overall isotopic mass balance must be preserved, the removal of variable amounts of ^13^C-depleted-carbon results in a shift of the isotopic composition of the remaining product, which in our case, was the CH_4_ gas^46^.

In conclusion, results of Trial 1 confirmed previous assumptions that CT from SL may be a tool for reducing in vitro CH_4_ production. However, using SL as sole source of feed to ruminal microorganisms might decrease digestibility of OM due to the high concentration of bioactive CT and to how this CT is presented (bound or unbound to protein and fiber-fractions). This draws into question the feasibility of using SL at greater proportions as a greenhouse gas mitigator. Trial 2 findings revealed that CT were degraded by ruminal microorganisms and underwent fractionation among the end products of fermentation, since it was possible to trace back the carbon that was present in the molecule of CT. This contrasts with the widely proposed paradigm that CT bypass the digestive tract of the ruminant undigested. Further research using isotopic composition coupled to methodologies that determine concentrations and changes in constitution of the CT molecule are needed to enhance understanding of CT utilization by ruminal microorganisms and the kinetics of CT in ruminants.

## Methods

Two trials were conducted at the North Florida Research and Education Center, from University of Florida, located in Marianna, FL, and followed all the procedures approved by the University of Florida Institutional Animal Care and Use Committee (Protocol #201810218). This study was conducted in accordance with ARRIVE guidelines.

### Trial 1

#### Donor animals, treatments, and sample preparation

Two ruminally-cannulated crossbred steers were kept under continuous stocking in a common bermudagrass [*Cynodon dactylon* (L.) Pers.] pasture monoculture with ad libitum access to bermudagrass hay and used as rumen fluid donors. Rumen fluid was collected in the morning (0700 h) of each sampling day, strained through four layers of cheesecloth, mixed in equal proportions, and kept under anaerobic conditions, by infusing CO_2_ into the rumen fluid vessel during all procedures^47^. All methods were carried out in accordance with relevant guidelines and regulations.

Treatments consisted of 100% bermudagrass hay (0SL), 50% bermudagrass hay and 50% sericea lespedeza hay (50SL), and 100% sericea lespedeza hay (100SL). Subsamples of hays were freeze-dried (FreeZone 6, LabConco, Kansas City, KC) and ground in a Wiley mill (Thomas Scientific, Swedesboro, NJ) to pass a 2-mm screen. Substrates were, then, mixed in their respective proportions on DM basis. Concentrations of CT were determined in freeze-dried samples of each of the substrates, as described by Kronberg et al.^9^ and Naumann et al.^42^. No hydrolysable tannins were detected in the SL hay or in bermudagrass hay. Chemical composition of substrates is described in Table 4.

**Table 4:** Chemical composition of substrates used in in vitro incubations of Trial 1.

Item	Substrates^a^		
	0SL	50SL	100SL
DM, g/kg	898	922	913
**Nutrient composition, g/kg DM**			
Ash	69	55	56
CP	85	108	161
NDF	747	389	377
ADF	345	323	258
Condensed tannins, g/kg DM	ND	36.3	82.3

ND, not detected.

^a^SL: sericea lespedeza [*Lespedeza cuneata* (Dum. Cours.) G. Don]; 0SL: 0% (fresh weight basis) sericea lespedeza hay (100% bermudagrass hay); 50SL: 50% sericea lespedeza hay + 50% bermudagrass hay; 100SL: 100% sericea lespedeza hay (0% bermudagrass hay).

#### Gas production, in vitro digestible organic matter and isotopic composition

A modified Tilley and Terry^48^ procedure was used to determine the in vitro organic matter digestibility. Preparations of samples and assay steps were followed according to Ciriaco et al.^49^. Four consecutive incubation runs were carried out with three flasks per substrate in addition to three blanks (no substrate).

Methane was measured separately from triplicate bottles incubated for each substrate, according to Ramin et al.^50^. For this, 0.7 g of the same substrates were incubated with 50 mL of a 3:1 McDougall’s buffer:ruminal fluid inoculum into 125-mL bottles fitted with rubber stoppers and crimp seal caps for 48 h under constant agitation (60 rpm) at 39 °C. At the end of each incubation, bottles were removed from incubator and gas pressure was measured in psi using a manometer (model YSB-068, BLD, China). Total gas produced was calculated according to Lopez et al.^51^. Four consecutive runs were carried out with three bottles per substrate. Triplicate bottles without substrate (blanks) were also included in each run to correct CH_4_ and GP for inoculum fermentation.

A subsample of gas was collected from each bottle headspace to determine concentration of CH_4_ and analyzed by gas chromatography (Agilent 7820A GC; Agilent Technologies, Palo Alto, CA). A flame ionization detector was used with a capillary column (Plot Fused Silica 25 m by 0.32 mm, Coating Molsieve 5A, Varian CP7536; Varian Inc. Lake Forest, CA). Injector, column, and detector temperatures were 80, 160, and 200 °C, respectively. Injector pressure was 20 psi with a total flow of 191.58 mL/min and a split flow of 185.52 mL/min with a 100:1 split ratio. Column pressure was 20 psi with a flow of 1.8552 mL/min. Detector makeup flow was 21.1 mL/min. The carrier gas was N_2_, and the run time was 3 min. Methane was calculated by multiplying total gas production (GP) per CH_4_ concentration.

For the δ^13^C-CH_4_ composition, freeze-dried samples were ball milled in a Mixer Mill MM 400 (Retsch, Newton, PA, USA) for 9 min at 25 Hz. Ball-milled samples were weighed (0.8 mg) in duplicate into tin capsules (solids “light” 5 × 9 mm, Thermo Fisher Scientific), and analyzed using an isotope ratio mass spectrometer (IsoPrime 100, Manchester, UK). The ^13^C/^12^C ratios are presented in the conventional delta (δ) notation, in per mil (‰) relative to the Pee Dee Belemnite (PDB).

#### Volatile fatty acids and ammonia nitrogen

The pH was recorded using a digital pH meter after opening the bottles. Concentrations of volatile fatty acids (VFA) in ruminal fluid samples were determined in a liquid–liquid solvent extraction using ethyl acetate^52^ and analyzed by gas chromatography (Agilent 7820A GC, Agilent Technologies, Palo Alto, CA) using a flame ionization detector and a capillary column (CP-WAX 58 FFAP 25 m 0.53 mm, Varian CP7767, Varian Analytical Instruments, Walnut Creek, CA). Column temperature was maintained at 110 °C, and injector and detector temperatures were 200 and 220 °C, respectively.

Concentrations of NH_3_-N in the rumen fluid were measured following the phenol-hypochlorite technique as described by Broderick and Kang^53^. Absorbance was read in 96-well, flat bottom plates at 620 nm using a plate reader (AccuSkan, Thermo Fisher Scientific, Waltham, MA).

### Trial 2

#### Plant establishment and enrichment

**Figure 3:**
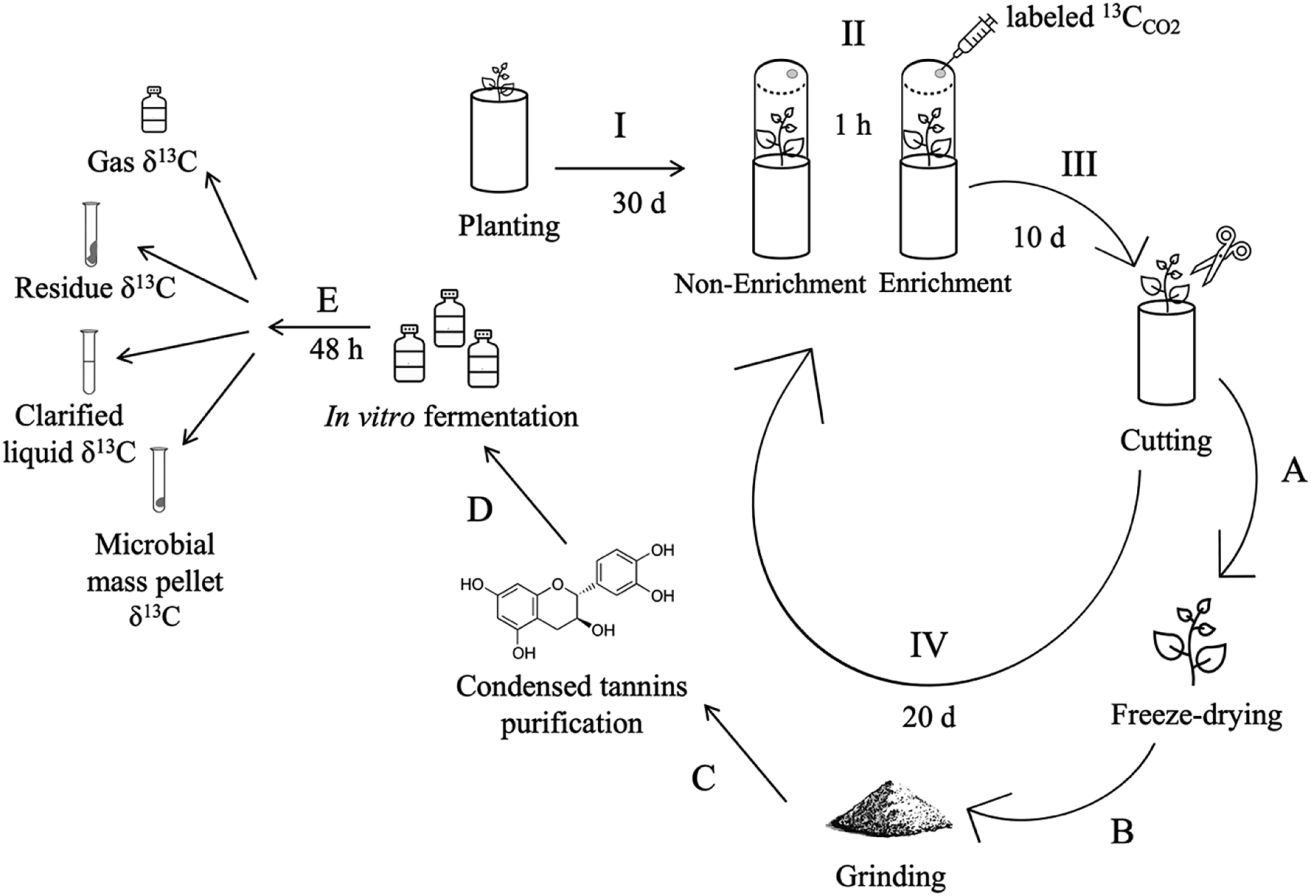
Depiction of Trial 2. Step I was carried out one time at the greenhouse. Steps II–IV were performed four times (experimental periods) at the greenhouse. Steps A–E were performed four times (experimental periods) at the laboratory.

Seedlings of ‘Au Grazer’ SL were obtained from FoxPipe Farm, in Laurens, SC. All methods were carried out in accordance with relevant guidelines and regulations. Seedlings averaged 15-cm height and were transplanted to 16 pots built out of PVC tubes (4.2 L) and kept in a greenhouse from August 2020 to February 2021. Pots were filled with 2.4 kg gravel and 3 kg 1:1 sand:potting mix (MiracleGro All Purpose Soil Mix, Marysville, OH). Eight pots were enriched with labeled ^13^C-CO_2_ (99% ^13^C, Aldrich, Darmstat, Germany) for 1 h, during 10 consecutive days and four experimental periods. Each experimental period totaled 30 days, comprised of 10 days of enrichment and 20 days of resting. First enrichment occurred 30 days after planting to allow plants to reach 30-cm height. For this, a 3-L translucid polyvinyl chamber fitted with a septum in the lid was tightly fitted to pots to cover plants for 1 h following the injection of 2 mL of ^13^C–CO_2_ with a 3-mL syringe. Enrichments started in the first hour after sunrise and temperature inside the chambers was monitored with a digital thermometer (AcuRite 00307 W, Chaney Instrument Co, Lake Geneva, WI) to not exceed 5 °C greater than greenhouse ambient. The morning after the last day of enrichment, plants were cut at 10-cm height, frozen, freeze-dried and ground to pass a 2-mm screen. The remaining eight pots were subjected to the same chamber handling, for 1 h, during 10 consecutive days, except that they were not enriched. Trial 2 steps are depicted in Fig. 3.

#### Condensed tannins purification

Subsamples of SL from each experimental period of enrichment were submitted to purification of the CT, according to Wolfe et al.^54^, Kronberg et al.^9^ and Naumann et al.^42^. As in Trial 1, concentrations of CT were determined in freeze-dried samples from SL, as described by Kronberg et al.^9^ and Naumann et al.^42^. No hydrolysable tannins were detected. Thirty grams of freeze-dried ground material was weighed into a 500-mL beaker to which was added 250 mL 70:30 (vol/vol) acetone:water. It was extracted for 30 min and filtered through disposable coffee paper filter to eliminate particulates. The filtrate was sequentially shaken three times with equal volume of ethyl ether for 45 s, until acetone solution was colorless, and the aqueous phase was collected. Subsequently, methanol:water (50:50 v:v) was mixed and filtered through Sephadex LH-20 (GE Healthcare Bio-Sciences Corp, Piscataway, NJ), which binds to the CT. Continuous washes with 70% acetone were made until the filtrate was colorless and all methanol:water was removed. The acetone was removed from the eluate by evaporation in the fume hood and the CT was frozen at -20 °C overnight and freeze-dried^42^.

#### In vitro incubations

For the in vitro incubations, treatments were pure CT Enriched, pure CT Not Enriched, Alfalfa, CT Enriched + Alfalfa, CT Not Enriched + Alfalfa, SL Enriched, and SL Not Enriched. Alfalfa was intentionally used as a non-tannin legume to provide enough nutrients and energy to ruminal microbes, and yet, present similar isotopic composition to SL, since CT as sole substrate could be a recalcitrant material and limit microbial attachment and fermentation. Twenty milligrams from each experimental substrate (20 of mono-substrate treatments or 10 of each in mixed substrates) were incubated with 50 mL of a 3:1 McDougall’s buffer:rumen fluid inoculum into 125-mL bottles fitted with rubber stoppers and crimp seal caps and kept under constant agitation (60 rpm) at 39 °C. Pressure in the bottles was constantly monitored using a manometer following recommendations by Theodorou^55^. After 48 h, gas from each bottle was collected and stored at room temperature until further analyses. Four consecutive incubation runs were carried out with three bottles per substrate in addition to three blanks (no substrate).

Bottles were placed on ice, opened, and pH was immediately recorded. The incubated rumen fluid components were separated by differential centrifugation and are referred to as end products of fermentation. Contents were centrifuged at 1000 × *g* for 10 min at 4 °C, the supernatant was transferred to a 50-mL centrifuge tubes, and the residue pellet stored at − 20 °C until further analyses. Supernatant from previous step was centrifuged at 20,000 × *g* for 20 min at 4 °C, and the new supernatant (clarified rumen fluid) was also stored at -20 °C until further analyses. The pellets from latter centrifugation were then washed three times with saline solution (0.9% NaCl) until the runoff was clear (subsequently referred to as microbial mass pellets).

The microbial mass pellets were frozen at − 20 °C overnight and freeze-dried. Subsequently, subsamples of residue pellet, clarified rumen fluid, and microbial pellets were weighed (~ 0.8 mg) in duplicate into tin capsules and analyzed using the IsoPrime isotope ratio mass spectrometer. The gas was analyzed using a Picarro gas isotopic analyzer (G2201-I, Picarro, Inc., Santa Clara, CA). The ^13^C/^12^C ratios are presented in the conventional delta (δ) notation, in per mil (‰) relative to the Pee Dee Belemnite (PDB).

Contribution of each substrate in the products of fermentation of mixed substrates were estimated using the equation below, as described by Jones et al.^56^:

$$\% {\text{C}}_{{3}} = {1}00{-}\left[ {{1}00 \times \left( {{\text{A}} - {\text{C}}} \right){/}\left( {{\text{B}} - {\text{C}}} \right)} \right]$$

where % C_3_ is the proportion of end-product of fermentation from CT intake; A is the δ^13^C of the sample from each product of fermentation; B is the δ^13^C of each product of fermentation from Alfalfa substrate; and C is the δ^13^C of each product of fermentation from CT substrates (enriched or not enriched).

### Statistical analyses

All data were analyzed as a randomized complete block design, using the Glimmix procedure of SAS (SAS Inst., Inc., Cary, NC; v.9.4), in which an average of the flasks (Trial 1) or the bottles (Trial 2) within run were considered the experimental unit, and the run was considered the block. Normality of distribution and homogeneity of variance were evaluated using the Univariate procedure of SAS. Models included the fixed effect of substrate and the random effect of run. Covariance structures were based upon the smallest Akaike Information Criterion value. In Trial 1, the linear effect of inclusion of SL and the quadratic effect of inclusion of SL were estimated using the coefficients for orthogonal polynomials and the SAS estimate procedure. In Trial 2, means were compared using the PDIFF adjusted by Tukey’s test. Significance was declared at 5%.

## Data Availability

All data generated or analyzed during this study are included in the article.

## References

[1] Marone D (2022). Specialized metabolites: Physiological and biochemical role in stress resistance, strategies to improve their accumulation, and new applications in crop breeding and management. Plant Physiol. Biochem..

[2] Fagundes GM (2020). Tannin-rich plants as natural manipulators of rumen fermentation in the livestock industry. Molecules..

[3] Koenig KM, Beauchemin KA (2018). Effect of feeding condensed tannins in high protein finishing diets containing corn distillers’ grains on ruminal fermentation, nutrient digestibility, and route of nitrogen excretion in beef cattle. J. Anim. Sci..

[4] Rivera-Méndez C, Plascencia A, Torrentera N, Zinn RA (2017). Effect of level and source of supplemental tannin on growth performance of steers during the late finishing phase. J. Appl. Anim. Res..

[5] Puchala R (2018). Effects of different levels of lespedeza and supplementation with monensin, coconut oil, or soybean oil on ruminal methane emission by mature Boer goat wethers after different lengths of feeding. J. Appl. Anim. Res..

[6] Mehansho H, Butler LG, Carlson DM (1987). Dietary tannins and salivary proline-rich proteins: Interactions, induction, and defense mechanisms. Ann. Rev. Nutr..

[7] Makkar HPS (2003). Effects and fate of tannins in ruminant animals, adaptation to tannins, and strategies to overcome detrimental effects of feeding tannin-rich feeds. Small Rumin. Res..

[8] Terrill TH, Waghorn GC, Woolley DJ, McNabb WC, Barr TN (1994). Assay and digestion of ^14^C-labelled condensed tannins in the gastrointestinal tract of sheep. Br. J. Nutr..

[9] Kronberg SL (2018). Effects of feeding Lespedeza cuneata pellets with Medicago sativa hay to sheep: Nutritional impact, characterization and degradation of condensed tannin during digestion. Anim. Feed Sci. Technol..

[10] Quijada J (2018). Condensed tannin changes along the digestive tract in lambs fed with sainfoin pellets or hazelnut skins. J. Agric. Food Chem..

[11] van Cleef FOS (2021). Methane emissions and δ^13^C composition from beef steers consuming increasing proportions of sericea lespedeza hay on bermudagrass hay diets. J. Anim. Sci..

[12] Porter LJ, Hrstich LN, Chan BC (1986). The conversion of procyanidins and prodelphinidins to cyanidin and delphinidin. Phytochemistry..

[13] Naumann HD, Tedeschi LO, Zeller WE, Huntley NF (2017). The role of condensed tannins in ruminant animal production: Advances, limitations and future directions. R. Bras. Zootec..

[14] Bird JA, van Kessel C, Horwath WR (2003). Stabilization of C^13^ carbon and immobilization of N^15^ nitrogen from rice straw in humic fractions. Soil Sci. Soc. Am. J..

[15] Prescott CE (2010). Litter decomposition: what controls it and how can we alter it to sequester more carbon in forest soils. Biogeochemistry..

[16] Soong JR (2014). Design and operation of a continuous 13C and 15N labeling chamber for uniform or differential, metabolic and structural, plant tissue isotope labeling. J. Vis. Exp..

[17] Bird JA, Torn MS (2006). Fine roots vs needles: A comparison of (13)C and (15)N dynamics in a ponderosa pine forest soil. Biogeochemistry..

[18] Gemeda BS, Hassen A (2015). Effect of tannin and species variation on in vitro digestibility, gas, and methane production of tropical browse plants. Asian Aust. J. Anim. Sci..

[19] Matthews C (2019). The rumen microbiome: A crucial consideration when optimising milk and meat production and nitrogen utilisation efficiency. Gut Microbes..

[20] Patra AK, Saxena J (2011). Exploitation of dietary tannins to improve rumen metabolism and ruminant nutrition. J. Sci. Food Agric..

[21] Tedeschi LO, Ramírez-Restrepo CA, Muir JP (2014). Developing a conceptual model of possible benefits of condensed tannins for ruminant production. Animal..

[22] Mueller-Harvey I (2018). Benefits of condensed tannins in forage legumes fed to ruminants: Importance of structure, concentration, and diet composition. Crop Sci..

[23] Tiemann TT (2008). Effect of the tropical tannin-rich shrub legumes *Calliandra calothyrsus* and *Flemingia macrophylla* on methane emission and nitrogen and energy balance in growing lambs. Animal..

[24] Hariadi BT, Santoso B (2010). Evaluation of tropical plants containing tannin on in vitro methanogenesis and fermentation parameters using rumen fluid. J. Sci. Food Agric..

[25] Beauchemin KA, McGinn SM, Martinez TF, McAllister TA (2007). Use of condensed tannin extract from quebracho trees to reduce methane emissions from cattle. J. Anim. Sci..

[26] Animut G (2008). Methane emission by goats consuming diets with different levels of condensed tannins from lespedeza. Anim. Feed Sci. Tech..

[27] Min BR, Barry TN, Atwood GT, McNabb WC (2003). The effect of condensed tannins on the nutrition and health of ruminants fed fresh temperate forages: A review. Anim. Feed Sci. Technol..

[28] Russell JB, Mantovani HC (2002). The bacteriocins of ruminal bacteria and their potential as an alternative to antibiotics. J. Mol. Microbiol. Biotechnol..

[29] Gurbuz Y (2009). Efectos del contenido de taninos condensados de algunas especies de leguminosas en la emisión de gas metano. Rev. Cubana Cienc. Agric..

[30] Van Soest PJ (1994). Nutritional Ecology of the Ruminant.

[31] Andries JI, Buysse FX, De Brabander DL, Cottyn BG (1987). Isoacids in ruminant nutrition: Their role in ruminal and intermediary metabolism and possible influences on performances—A review. Anim. Feed Sci. Technol..

[32] Vargas LH (2001). Influência de Rumensin®, óleo de soja e níveis de concentrado sobre o consumo e os parâmetros fermentativos ruminais em bovinos. Rev. Bras. de Zootec..

[33] Mahachi LN (2020). Sericea lespedeza (Lespedeza juncea var. sericea) for sustainable small ruminant production: Feed, helminth suppressant and meat preservation capabilities. Anim. Feed Sci. Technol..

[34] Mueller-Harvey I (2006). Unravelling the conundrum of tannins in animal nutrition and health. J. Sci. Food Agric..

[35] Bhatta R, Saravanan M, Baruah L, Prasad CS (2015). Effects of graded levels of tannin-containing tropical tree leaves on *in vitro* rumen fermentation, total protozoa, and methane production. J. Appl. Microbiol..

[36] Agle M (2010). The effects of ruminally degraded protein on rumen fermentation and ammonia losses from manure in dairy cows. J. Dairy Sci..

[37] DeNiro MJ, Epstein S (1978). Influence of diet on the distribution of carbon isotopes in animals. Geochim. Cosmochim. Acta..

[38] Gowik W, Westhoff P (2011). The path from C_3_ to C_4_ photosynthesis. Plant Physiol..

[39] Coleman, S. W., Moore, J. E., & Wilson, J. R. Quality and utilization. In: *Warm-Season (C4) Grasses* (Moser, L. E., Burson, B. L., Sollenberger, L. E., eds.) 267–308 (2004).

[40] Makkar HPS, Becker K, Abel H, Szegletti C (1995). Degradation of condensed tannins by rumen microbes exposed to quebracho tannins (QT) in rumen simulation technique (RUSITEC) and effects of QT on fermentative processes in the RUSITEC. J. Sci. Food Agric..

[41] Perez-Maldonado RA, Norton BW (1996). Digestion of 14C-labelled condensed tannins from *Desmodium intortum* in sheep and goats. Br. J. Nutr..

[42] Naumann HD, Sepela R, Rezaire A, Masih SE, Zeller WE, Reinhardt LA, Robe JT, Sullivan ML, Hagerman AE (2018). Relationships between structures of condensed tannins from Texas legumes and methane production during in vitro rumen digestion. Molecules..

[43] Carre B, Brillouet JM (1986). Yield and composition of cell wall residues isolated from various feedstuffs used for non-ruminant farm animals. J. Sci. Food Agric..

[44] Distel RA, Provenza FD (1991). Experience early in life affects voluntary intake of blackbrush by goats. J. Chem. Ecol..

[45] Bayle K, Akoka S, Remaud GS, Robins RJ (2015). Nonstatistical 13C distribution during carbon transfer from glucose to ethanol during fermentation is determined by the catabolic pathway exploited. J. Biol. Chem..

[46] Hobbie EA, Werner RA (2004). Intramolecular, compound-specific, and bulk carbon isotope patterns in C3 and C4 plants: A review and synthesis. New Phytol..

[47] Grant RJ, Mertens DR (1992). Development of buffer systems for pH control and evaluation of pH effects on fiber digestion in vitro. J. Dairy Sci..

[48] Tilley JMA, Terry RA (1963). A two-stage technique for the in vitro digestion of forage crops. J. Brit. Grassland Soc..

[49] Ciriaco FM (2021). Ruminal in situ degradability of forage components and in vitro organic matter digestibility of warm-season grasses treated with calcium oxide. Transl. Anim. Sci..

[50] Ramin M, Krizsan SJ, Jančík F, Huhtanen P (2013). Measurements of methane emissions from feed samples in filter bags or dispersed in the medium in an in vitro gas production system. J. Dairy Sci..

[51] Lopez S (2007). Some methodological and analytical considerations regarding application of the gas production technique. Anim. Feed. Sci. Technol..

[52] Ruiz-Moreno M, Binversie E, Fessended SW, Stern MD (2015). Mitigation of in vitro hydrogen sulfide production using bismuth subsalisylate with and without monensin in beef feedlot diets. J. Anim. Sci..

[53] Broderick GA, Kang JH (1980). Automated simultaneous determination of ammonia and total amino acids in ruminal fluid and in vitro media. J. Dairy Sci..

[54] Wolfe RM, Terrill TH, Muir JP (2008). Drying method and origin of standard affect condensed tannin (CT) concentrations in perennial herbaceous legumes using simplified butanol-HCl CT analysis. J. Sci. Food Agric..

[55] Theodorou MK, Williams BA, Dhanoa MS, McAllan AB, France J (1994). A simple gas production method using a pressure transducer to determine the fermentation kinetics of ruminant feeds. Anim. Feed. Sci. Technol..

[56] Jones RJ, Ludlow MM, Throughton JH, Blunt CG (1979). Estimation of the proportion of C3 and C4 plant species in the diets of animals from the ratio of natural ^12^C and ^13^C isotopes in the faeces. J. Agric. Sci..

